# IL-7 Receptor Recovery on CD8 T-Cells Isolated from HIV+ Patients Is Inhibited by the HIV Tat Protein

**DOI:** 10.1371/journal.pone.0102677

**Published:** 2014-07-17

**Authors:** Elliott M. Faller, Mark J. McVey, Paul A. MacPherson

**Affiliations:** 1 Ottawa Hospital Research Institute, Chronic Disease, Ottawa, Ontario, Canada; 2 Division of Infectious Diseases, Ottawa Hospital General Campus, Ottawa, Ontario, Canada; 3 Faculty of Medicine, Department of Biochemistry, Microbiology and Immunology, University of Ottawa, Ottawa, Ontario, Canada; Istituto Superiore di Sanità, Italy

## Abstract

Expression of the IL-7 receptor α-chain (CD127) is decreased on CD8 T-cells in HIV infected patients and partially recovers in those receiving antiretroviral therapy with sustained viral suppression. We have shown that soluble HIV Tat protein down regulates CD127 expression on CD8 T-cells isolated from healthy HIV-negative individuals. Tat is taken up by CD8 T-cells via endocytosis, exits the endosome and then translocates to the inner leaflet of the cell membrane where it binds to the cytoplasmic tail of CD127 inducing receptor internalization and degradation by the proteasome. This down regulation of CD127 by Tat results in impaired CD8 T-cell function. Interestingly, suppression of CD127 by Tat is reversible and requires the continual presence of Tat in the culture media. We thus questioned whether the low IL-7 receptor expression evident on CD8 T-cells in HIV+ patients was similarly reversible and if suppression of the receptor could be maintained *ex vivo* by Tat protein alone. We show here that when CD8 T-cells isolated from HIV+ patients are incubated alone in fresh medium, low CD127 expression on the cell surface recovers to normal levels. This recovery of CD127, however, is completely inhibited by the addition of HIV Tat protein to the culture media. This study then provides evidence that soluble factor(s) are responsible for low CD127 expression on circulating CD8 T-cells in HIV+ individuals and further implicates Tat in suppressing this receptor essential to CD8 T-cell proliferation and function.

## Introduction

Impaired cell mediated immunity is the clinical hallmark of HIV infection and is directly responsible for the appearance of many opportunistic infections in patients with progressive disease. In vitro studies have confirmed functional deficits in CD8 T-cells isolated from HIV+ individuals including reduced proliferation and impaired cytolytic activity. [1], [2], [3], [4] Indeed, both HIV− and EBV-specific CD8 T-cells can be found in the circulation at relatively normal frequencies in HIV-infected patients with advanced disease [5], [6], [7], [8], [9] yet these cells respond poorly to their cognate antigens and fail to express normal levels of perforin and interferon (IFN)-γ, or demonstrate effective cytolytic activity. [6], [9], [10], [11], [12], [13] This is of obvious advantage to HIV as by disarming cell mediated immunity the virus is able to avoid elimination and establish chronic infection.

Interleukin (IL)-7 is essential for normal T-cell development and function. In addition to playing a critical role in peripheral immune homeostasis [9], [14], [15], [16], [17], [18] and the development and maintenance of T-cell memory, [19], [20] IL-7 also plays an important role in the activation of CD8 T-cells in response to foreign antigen. IL-7 independently stimulates CD8 T-cell proliferation, [21], [22], [23], [24] and potentiates cytolytic activity [25], [26], [27], [28], [29], [30], [31] by enhancing production of IFN-γ following TCR stimulation [32], [33] and by inducing accumulation of intracellular perforin. [34], [35] Given the important role IL-7 plays in CD8 T-cell responses, decreased IL-7 signaling would be expected to result in impaired cell mediated immunity and inefficient control of viral pathogens including HIV.

IL-7 signaling occurs via its receptor, a heterodimer composed of a unique α-chain (CD127) [36] and the common γ-chain (CD132). [37] We and others have shown decreased expression of the IL-7R α-chain on CD8 T-cells in HIV-infected individuals with uncontrolled viral replication [38], [39], [40], [41], [42], [43], [44] and partial recovery in patients receiving highly active antiretroviral therapy with sustained viral suppression. [38], [41] Notably, the decrease in CD127 expression in HIV+ individuals correlates with impaired CD8 T-cell responses. Vingerhoets *et al* [45] found compared to controls CD8 T-cells from HIV+ individuals with low CD127 expression were less able to form blasts and up regulate CD25 in response to IL-7. Ferrari *et al* [46] also found anti-HIV CD8 T-cells isolated from patients with advanced disease could not be expanded *in vitro* following stimulation with HIV antigens and IL-7. Thus it appears decreased CD127 expression leads to impaired CD8 T-cell proliferation and function and thus may contribute to reduced cell mediated immunity in HIV+ patients.

The factors responsible for down regulating CD127 during HIV infection have yet to be definitively established. Notably, decreased CD127 expression has been observed on all CD8 T-cell subsets in HIV+ individuals including resting naïve cells with concomitant low CD38 expression suggesting suppression of this receptor may not be the result of chronic T-cell activation. [38], [39], [42], [47] Several soluble factors likely play a role and we have previously shown soluble HIV Tat protein specifically down regulates CD127 on the surface of CD8 T-cells isolated from healthy HIV-negative volunteers. [35], [48] Tat, a small 15 kdal viral polypeptide, is secreted by infected CD4+ cells [49], [50], [51], [52], [53], [54], [55] and is rapidly internalized by neighboring uninfected lymphocytes [51], [56], [57] through clathrin-coated pits. [58] Once inside the cell, Tat exits late endosomes upon the usual acidification of these vesicles [58], [59] and translocates to the inner leaflet of the plasma membrane where it binds to the cytoplasmic tail of CD127. [48] This interaction with Tat induces receptor aggregation and removal from the cell surface through a process dependent on microtubules and directs CD127 to the proteasome for degradation. [48] The effect of Tat on CD127 expression is both dose and time dependent, can be blocked with anti-Tat antibodies, [35] and occurs in the presence of cycloheximide indicating a direct effect and that new protein synthesis is not required [48]. Tat down regulates CD127 equally on both naïve and memory CD8 T-cells yet has no effect on a number of other cell surface proteins including CD25, CD28 and CD56 indicating a stable CD8 T-cell phenotype and lack of nonspecific activation in the presence of soluble Tat protein. Tat also has no effect on the expression of CD132, the common γ-chain that associates with CD127 to form the IL-7 receptor. Importantly, Tat-induced down regulation of CD127 results in deficits in CD8 T-cell activity. Pre-incubating CD8 T-cells with Tat inhibits proliferation and accumulation of intra-cellular perforin following stimulation with IL-7. [35] Thus, by down regulating CD127 expression on CD8 T-cells, soluble HIV Tat protein is able to decrease IL-7 signaling and impair both CD8 T-cell expansion and cytolytic capacity. Interestingly, Tat-induced down regulation of CD127 on the surface of CD8 T-cells is reversible and requires the continual presence of this viral protein. When Tat is removed from the culture media, CD127 returns to normal levels with 24 hours. [35].

In view of the fact that soluble Tat protein is able to down regulate CD127 on CD8 T-cells and that this down regulation is reversible after up to 72 hours in culture upon removal of Tat from the media, we asked whether CD127 expression could recover on CD8 T-cells isolated from HIV+ patients and if so if this recovery is inhibited by Tat.

## Materials and Methods

### Ethics Statement

This work was reviewed and approved by the Ottawa Hospital Research Ethics Board and informed written consent was obtained from all participants. No children were used in this study. Written consent was obtained from all participants after reading a 5 page document outlining the phlebotomy procedure, study outline and research goals.

### Patients

Patients were recruited from The Ottawa Hospital Immunodeficiency Clinic according to the following inclusion criteria: age over 18 years; naïve to antiretroviral therapy or off therapy for at least three months; a blood plasma HIV viral load above detection (>50 copies/ml); CD4 T-cell count >200 cells/µl; no co-morbidities; and able to provide signed informed consent. Exclusion criteria included co-infection with hepatitis B and/or hepatitis C virus; and clinical or laboratory evidence of an active infection or malignancy. CD4 and CD8 T-cell counts, percentages and ratios as well as HIV viral loads were obtained from the patients’ medical records.

### Reagents

Purified HIV-1 Tat protein (86 amino acids) was purchased from Advanced Bioscience Laboratories Inc. (Kensington, MD). Lyophilized protein was resuspended to 1 mg/ml in phosphate buffered saline (PBS) containing 1 mg/ml bovine serum albumin (BSA) and 0.1 mM dithiothreitol. Tat protein is reportedly >95% pure by heparin-affinity chromatography and reverse phase high performance liquid chromatography. Anti-CD8-phycoerythrin-Cy5 (PC5) (B9.11) and anti-CD127-phycoerythrin (PE) (R34.34) fluorochrome-labeled monoclonal antibodies were purchased from Immunotech Beckman Coulter (Marseille, France). All fluorochrome labeled antibodies were titrated and used at saturating concentrations.

### Cell Purification and Culture

Patient blood was drawn into sodium heparin-containing tubes and processed within 2 hours. Peripheral blood mononuclear cells (PBMC) were isolated by Ficoll-Paque density centrifugation followed by CD8 T-cell purification using the MACS Microbead CD8+ Cell AutoMACS Isolation System (Miltenyi Biotec, Auburn, CA). By this method, cell purity was consistently >95% CD8+. Purified CD8 T-cells were incubated at 1×10^6^ cells/ml in media comprised of RPMI 1640 (Hyclone, Logan, UT) supplemented with 20% fetal calf serum (FCS; Cansera, Rexdale, ON, Canada) plus 100 U/ml penicillin, 100 µg/ml streptomycin and 0.2 M L-glutamine (RPMI-20). All cultures were maintained in a humidified incubator at 37°C in the presence of 5% CO_2_.

### Flow cytometry

At times indicated, cells were incubated with anti-CD8-PC5 and anti-CD127-PE fluorochrome-labeled antibodies for 30 minutes in the dark at room temperature, and then analyzed by flow cytometry using a Coulter Epics ALTRA flow cytometer (Fullerton, CA). Live cells were gated on the basis of side and forward scatter and at least 10,000 events were recorded for each sample. Isotype controls were performed for each fluorochrome-conjugated antibody. Resulting profiles were analyzed with De Novo FCS Express 2 software (Los Angeles, CA). As demonstrated previously, decreased detection of CD127 by flow cytometry using the R34.34 antibody correlates with reduced CD127 protein as determined by Western blot analysis [48], [60].

### Statistical Analysis

Statistical comparisons were carried out using a two-tailed, paired student t-test with 95% confidence intervals and p values given throughout the text reflect this analysis. Given the sample sizes for the two patient groups, parallel nonparametric analyses were also carried out using the Wilcoxon matched pair test again with 95% confidence intervals. Statistical significance as defined by a p value greater or less than 0.05 remained the same for all comparisons irrespective of parametric or nonparametric analysis.

## Results

### Patients

Patient characteristics and demographics (N = 11) are shown in Table 1. The mean age was 39 years (range 21–56) and all were men. The mean CD4 T-cell count was 355 cells/µl or 23% (range 203–573; 13–41%) with a mean CD8 T-cell count of 1043 cells/µl or 60% (range 246–2411; 37–73%). The average CD4/CD8 T-cell ratio was 0.41. HIV viral loads ranged from 484 to 500,000 copies/ml with a mean of 94,023 copies/ml. Nine patients had never received antiretroviral therapy and 2 had been previously treated but had discontinued therapy five months prior to enrolment. The patients were otherwise in good health at the time of analysis.

**Table 1:** Study patients.

Participant	Age	Sex	CD3CD4 count (%)	CD3CD8 count (%)	CD4/CD8 ratio	Viral load (copies/ml)	Prior Rx
1	42	M	203 (16)	817 (64)	0.25	13,510	No
2	21	M	247 (17)	995 (69)	0.25	7,953	No
3	39	M	271 (18)	967 (65)	0.28	105,425	Yes
4	30	M	474 (28)	932 (54)	0.51	22,051	No
5	44	M	344 (22)	923 (59)	0.37	500,000	No
6	56	M	390 (24)	868 (53)	0.45	75,216	No
7	24	M	272 (41)	246 (37)	1.1	61,517	No
8	52	M	359 (21)	1091 (64)	0.3	484	No
9	43	M	530 (33)	850 (53)	0.6	27,158	No
10	43	M	573 (16)	2411 (66)	0.2	109,771	Yes
11	41	M	248 (13)	1376 (73)	0.2	111,174	No
Average	39		355 (23)	1043 (60)	0.41	94,023	

Patients were analyzed in two separate groups, patients 1–7 (assayed at isolation and after 24 hours) and patients 6–11 (measured at isolation and every 24 hours over 72 hours). The groups were not statistically different in any characteristic analyzed including age, T-cell counts/percentages, or HIV viral loads (all p values ≥0.24).

### CD127 recovers ex vivo on CD8 T-cells isolated from HIV+ individuals

**Figure 1:**
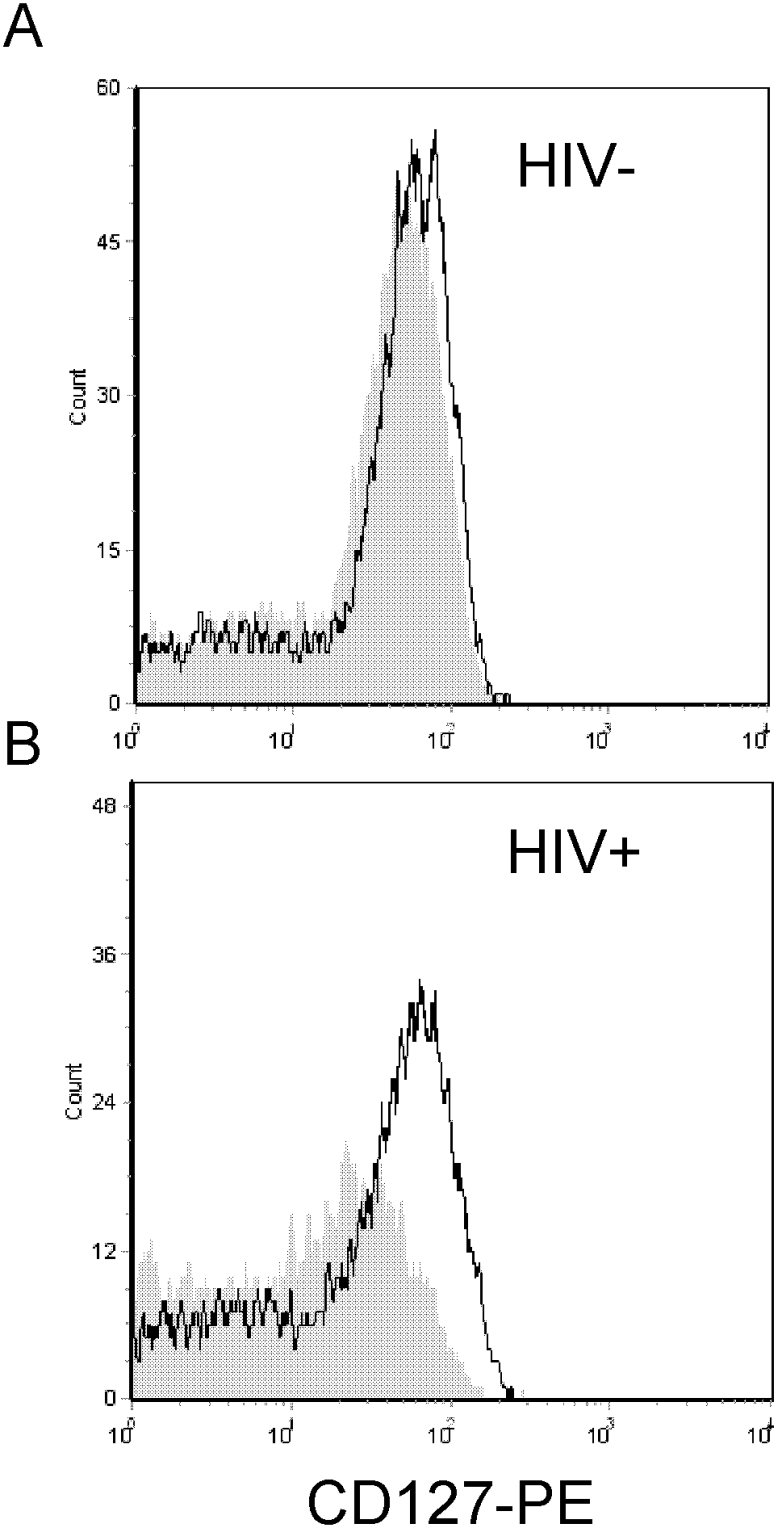
CD127 recovers ex vivo to normal levels on CD8 T-cells isolated from an HIV-infected individual. CD127 expression on purified CD8 T-cells is shown immediately following cell isolation (gray fill) and after 24 hours in culture medium (black line). Two representative histograms are shown, one from a healthy HIV-negative volunteer (panel A) and one from an HIV-infected patient with a CD4 count of 359 cells/µl and an HIV viral load (VL) of 484 copies/ml (panel B).

**Figure 2:**
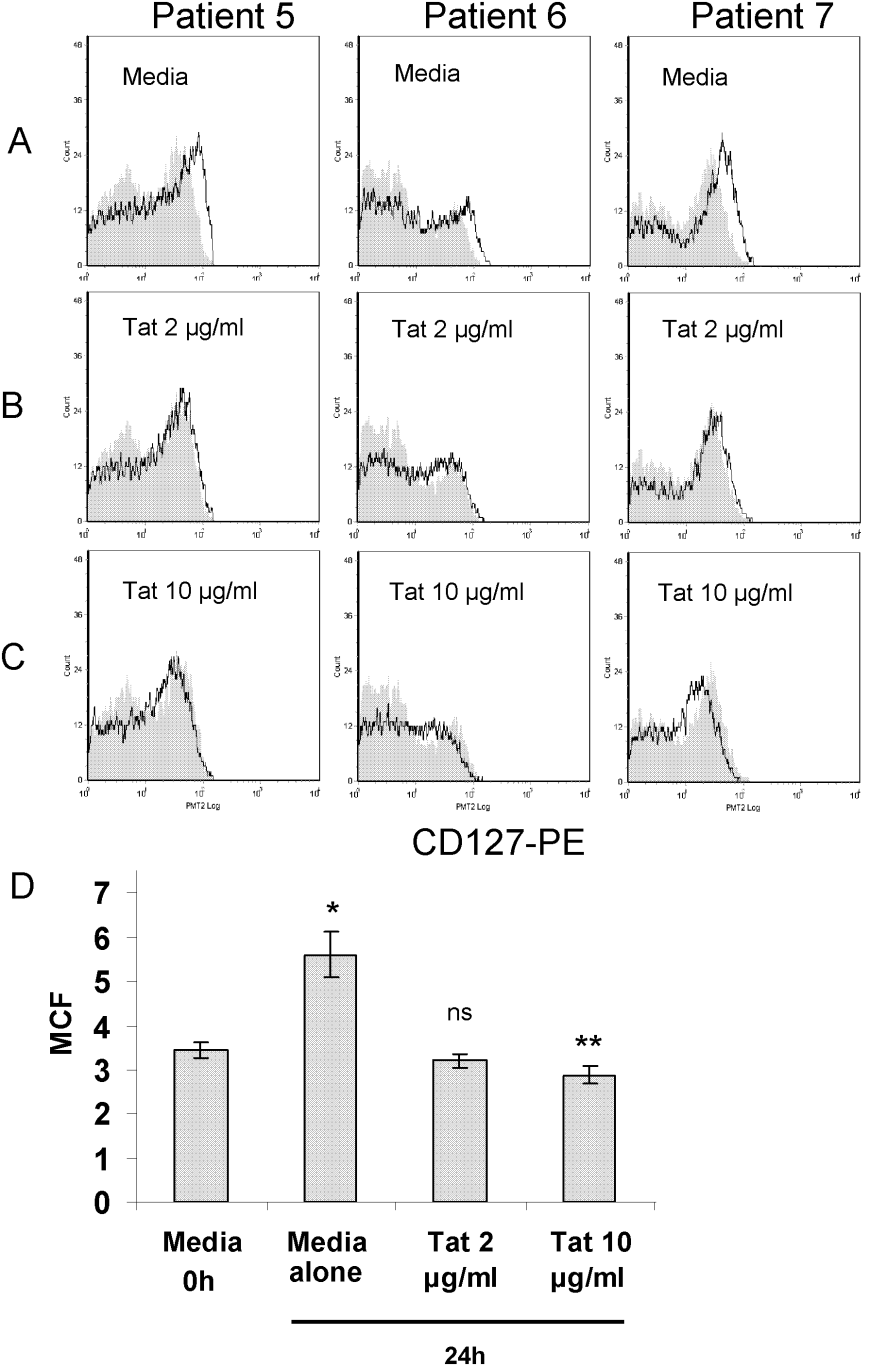
While CD127 expression recovers ex vivo on CD8 T-cells isolated from HIV+ patients, soluble HIV Tat protein maintains suppression of the receptor. CD8 T-cells isolated from HIV-positive patients were cultured in media alone (panel A) or in media containing purified Tat protein at 2 µg/ml (panel B) or 10 µg/ml (panel C) for 24 hours. Three representative HIV-positive patients are shown (Patient 5: CD4 = 344 cells/µl and VL = 500,000 copies/ml; Patient 6: CD4 = 390 cells/µl and VL = 75,216 copies/ml; Patient 7: CD4 = 272 cells/µl and VL = 61,517 copies/ml). CD127 expression on purified CD8 T-cells is shown immediately following cell isolation (gray fill) and after 24 hours in culture (black line). Panel D: Composite data from 7 patients showing changes in CD127 mean channel fluorescence. CD8 T-cells isolated from HIV-positive patients were cultured in media alone, or in media containing purified Tat protein at 2 µg/ml or 10 µg/ml for 24 hours. CD127 mean channel fluorescence increased on cells maintained in media alone for 24 hours compared to the time of isolation (*p = 0.005), remained unchanged in the presence of 2 µg/ml soluble Tat protein (ns), and decreased slightly when cultured in the presence of 10 µg/ml Tat (**p = 0.007).

CD127 is down regulated on CD8 T-cells in HIV+ patients and partially recovers following viral suppression with highly active antiretroviral therapy. [38], [39], [40], [41], [42], [43] We have previously shown soluble HIV Tat protein down regulates CD127 on healthy CD8 T-cells in culture [35] and that this down regulation is reversible with CD127 recovering back to normal levels once Tat is removed from the culture media. This prompted us to ask whether the suppression of CD127 on CD8 T-cells in HIV+ individuals is reversible and whether these cells could re-express CD127 when maintained in culture media *ex vivo*. To address this, CD8 T-cells were isolated from HIV-infected patients not on therapy and a portion of the cells were analyzed immediately for CD127 expression by flow cytometry. The remainder of the cells were washed in PBS and incubated for up to 72 hours in RPMI-20, and CD127 expression was monitored by flow cytometry at 24 hour intervals. Figure 1 shows typical flow histograms demonstrating CD127 expression on CD8 T-cells from an HIV-negative individual (panel A) and from an HIV+ individual (panel B) immediately following CD8 T-cell isolation (grey fill) and after 24 hours in culture media (black line). Consistent with previous reports, CD127 expression was significantly decreased on CD8 T-cells from the HIV+ individual immediately following isolation. However, when purified CD8 T-cells from this same individual were maintained *ex vivo* in RPMI-20, CD127 recovered over 24 hours to levels equivalent to that seen in the HIV-negative control. This was demonstrated in both percent positive staining cells and in mean channel fluorescence. In comparison, CD127 expression increased only marginally on CD8 T-cells isolated from the HIV-negative individual. Figure 2 shows similar data as both histograms for three representative HIV+ patients (panel A) and in composite form for seven patients (patients 1–7; panel D). In all cases, CD127 expression increased within 24 hours on CD8 T-cells isolated from HIV+ individuals when cultured in media alone (figure 2 panel A; grey fill, immediately following isolation; black line, after 24 hours in culture media). Indeed, the average mean channel fluorescence for CD127 staining on CD8 T-cells increased from 3.45+/−0.19 (mean +/− standard error of the mean) at the time of isolation to 5.59+/−0.61 after 24 hours in culture media (p = 0.005). We therefore conclude that suppression of CD127 on CD8 T-cells in HIV+ individuals is reversible and increases over 24 hours when the cells are maintained in fresh medium *ex vivo*. This suggests a soluble factor or factors are responsible for the down regulation of CD127 *in* *vivo*.

### HIV Tat protein maintains suppression of CD127 on CD8 T-cells isolated from HIV+ patients

In view of our previous findings demonstrating soluble HIV Tat protein specifically down regulates CD127 on the surface of CD8 T-cells isolated from healthy volunteers, we questioned whether this viral protein alone could maintain suppression of the IL-7 receptor on CD8 T-cells isolated from HIV+ individuals. To address this, CD8 T-cells were purified from HIV+ patients as described and incubated in either RPMI-20 alone or in media containing purified Tat protein. Figure 2 shows flow histograms from three representative patients (panels B and C) and composite data from patients 1–7 (panel D). In contrast to cells incubated in medium alone, when the cells were cultured in the presence of soluble Tat protein, suppression of CD127 was maintained. Indeed, in the presence of 2 µg/ml Tat the expression of CD127 on CD8 T-cells remained stable (figure 2, panel B) with an average mean channel fluorescence of 3.20+/−0.21, essentially unchanged compared to the levels of CD127 at the time of isolation (p = 0.39; panel D). Interestingly, higher concentrations of Tat protein (10 µg/ml) were able to further suppress CD127 expression albeit slightly after 24 hours in culture (figure 2, panel C). Under these conditions, the average CD127 mean channel fluorescence decreased to 2.63+/−0.19 (p = 0.007 compared to time of isolation; panel D).

**Figure 3:**
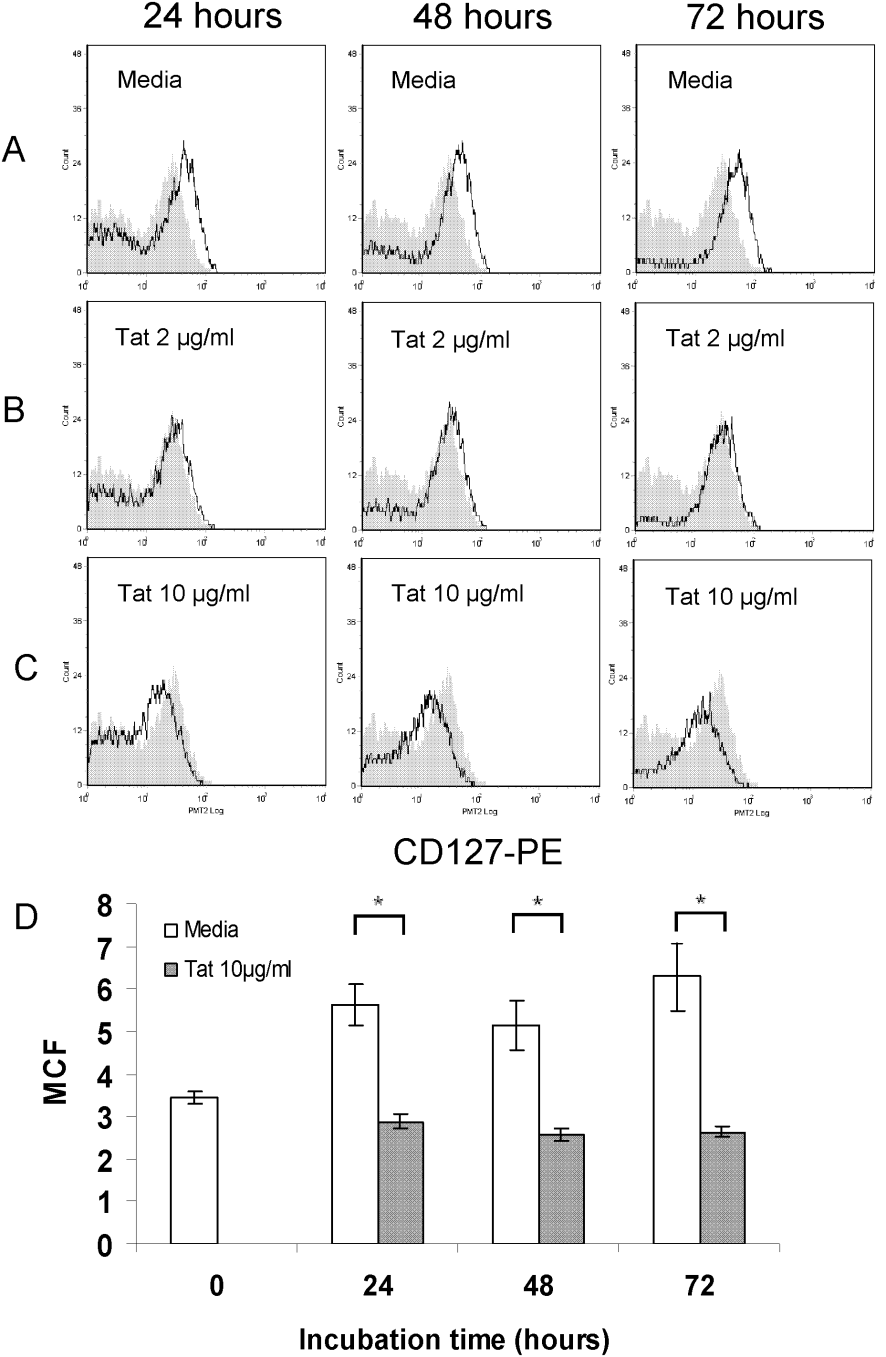
Soluble HIV Tat protein maintains suppression of CD127 expression on CD8 T-cells isolated from HIV-infected individuals over at least 72 hours. Representative histograms show CD127 expression on CD8 T-cells isolated from an HIV-infected patient (Patient 7: CD4 = 272 cells/µl and VL = 61,517 copies/ml) and incubated in media alone (panel A) or in media containing 2 µg/ml (panel B) or 10 µg/ml (panel C) soluble Tat protein. CD127 expression on purified CD8 T-cells is shown immediately following cell isolation (gray fill) and after 24, 48 and 72 hours in culture (black lines). Panel D: Composite data from 6 patients showing changes in CD127 mean channel fluorescence. CD8 T-cells were isolated from HIV-infected patients and incubated in media alone, or in media containing 10 µg/ml Tat protein for 72 hours. For cells cultured in media alone, CD127 mean channel fluorescence increased within 24 hours and was maintained thereafter (p≤0.01 at 24, 48 and 72 hours compared to time of isolation). CD127 expression was not statistically different comparing cells cultured in media for 48 and 72 hours compared to 24 hours (p = 0.70 and 0.98 respectively). When cells were cultured in the presence of Tat protein, CD127 expression remained low with mean channel fluorescence readings significantly lower compared to cells cultured in media alone. (*indicates p≤0.003.).

To more fully describe the dynamics of CD127 recovery and its suppression by Tat, CD8 T-cells isolated from six HIV+ individuals (participants 6–11) were followed over 72 hours. Figure 3 shows flow histograms from one representative patient (Patient 7) while composite data for all six patients are shown in panel D. While time to peak recovery varied somewhat from patient to patient, CD127 expression increased on purified CD8 T-cells isolated from HIV+ individuals when cultured in media alone and was maintained at levels comparable to HIV-negative controls for over 72 hours (figure 3, panel A). Consistent with the data above, the average CD127 mean channel fluorescence increased from 3.52+/−0.19 at the time of isolation to 6.35+/−0.54 after 24 hours in culture media (p = 0.0003), and then remained stable (panel D). When CD8 T-cells were cultured in media containing purified Tat protein, suppression of CD127 was fully maintained for up to at least 72 hours (figure 3, panels B, C and D). In the presence of 10 µg/ml Tat, CD127 mean channel fluorescence remained less than 3.2 over the 72 hours and well below recovered CD127 levels on cells maintained in parallel in medium alone (p≤0.003).

## Discussion

This work addresses two questions. First, we asked whether the low CD127 expression on CD8 T-cells in HIV+ patients is reversible. Several groups including our own have reported an increase in CD127 on the surface of CD8 T-cells in HIV+ patients following viral suppression with effective antiretroviral therapy. [38], [39], [40], [41], [42], [43] However, it is not clear whether this recovery is due to an increase in CD127 on existing T-cells or reflects a repopulation of new cells with higher CD127 expression during immune reconstitution. Although a contribution from the latter cannot be ruled out, we show here that suppression of CD127 on CD8 T-cells from HIV+ individuals is in fact reversible and that CD127 receptor density increases on existing cells *ex vivo*. The fact that CD8 T-cells from HIV+ patients are able to re-express CD127 once purified and placed in culture medium indicates reduced expression of the IL-7 receptor *in vivo* is the result of active suppression by soluble factors in the patient serum and not due to permanent alterations in the cells themselves. Similar recovery of CD127 on CD8 T-cells in mixed PBMC cultures from HIV+ patients has also been reported by Colle *et al*. [41] In their study, CD127 expression increased significantly after 3 days in culture on all CD8 T-cell subsets and most notably on naïve, central memory and effector memory cells. Rethi *et al*. [43] failed to detect CD127 recovery *ex vivo* but in their study they reported only a stable percentage of low/negative T-cells and therefore may have missed an increase in receptor expression since they did not measure changes in mean channel fluorescence on CD127+ cells.

We have previously shown soluble HIV Tat protein specifically down regulates CD127 on the surface of CD8 T-cells isolated from healthy HIV-negative volunteers. [35] Tat is secreted by HIV-infected cells [49], [51], [54] and can be detected in the media during *in vitro* infection [51], [52] as well as in the tissues and serum of HIV-infected patients. [53], [61] As noted in our previous study, Tat-induced suppression of CD127 is maintained only if Tat is continuously present in the media. When Tat is removed from the media, CD127 recovers on the cell surface to pre-treatment levels within 24 hours. [35] In view of this and the fact that soluble factors appear to actively suppress CD127 expression in vivo, we next asked whether soluble Tat protein alone could maintain suppression of CD127 *ex vivo* on CD8 T-cells isolated from HIV+ patients. We show here this is in fact the case. In all patients examined, CD127 uniformly remained low on CD8 T-cells isolated from HIV+ individuals when cultured in the presence of soluble Tat protein and essentially unchanged compared to the time of isolation. These data suggest soluble Tat protein may well play an active role in suppressing IL-7 receptor expression on CD8 T-cells *in vivo*.

Interestingly, lower concentrations of Tat are required to maintain suppression of CD127 on CD8 T-cells isolated from HIV+ individuals compared to concentrations needed to down regulate this receptor *de novo* on CD8 T-cells isolated from healthy HIV-negative volunteers. In a previous study we found 2 µg/ml of Tat induce a 4+/−1% decrease in CD127 expression on healthy CD8 T-cells while 10 µg/ml induced a 39+/−3% decline compared to cells maintained in media alone. [62] In the present study, Tat at 2 µg/ml was able to maintain full suppression of CD127 *ex vivo* on cells isolated from HIV+ patients. That lower concentrations are required to maintain receptor suppression is not surprising given the effects of Tat on CD127 are stoichiometric. Once taken up by CD8 T-cells, Tat interacts directly with the cytoplasmic tail of CD127 to induce receptor internalization and degradation. [48] The direct protein-protein interaction between Tat and CD127 necessary for receptor down regulation mandates higher Tat concentrations in healthy CD8 T-cells where the density of CD127 on the cell surface is much higher, whereas lower Tat concentrations could be sufficient to prevent re-accumulation of already low levels of CD127 on the surface of cells isolated from HIV+ patients.

Tat concentrations in the sera of HIV+ patients have been reported ranging 40–550 ng/ml, [53] some 4 to 250-fold lower than the concentrations used in our experiments. However, direct comparisons of *in vivo* protein concentrations to those used in *in vitro* assays should be approached with caution. For example, the Tat protein used in our assays is recombinant protein purified from E. coli. As such, recombinant proteins do not undergo post-translational modification and it is unlikely 100% of the purified protein retains biological activity. Thus *in vivo* concentrations of post-translationally modified Tat secreted from neighbouring HIV-infected CD4 cells and *in vitro* concentrations of recombinant Tat purified in the laboratory from E. coli may not be comparable. Perhaps more importantly the concentration of free Tat protein circulating in the sera of HIV infected patients likely does not reflect the levels of soluble Tat protein that cells are exposed to *in vivo*. Indeed, Tat concentrations in the peripheral circulation are likely quite low compared to concentrations in the lymph nodes and gut mucosa where the majority of HIV-infected T-cells reside.

Accumulating evidence indicates soluble Tat protein plays a significant role in the immune dysregulation evident in progressive HIV infection We have shown down regulation of CD127 by Tat results in impaired CD8 T-cell function including reduced proliferation and decreased accumulation of intracellular perforin following stimulation with IL-7. [35] Others have also shown lymphocytes exposed to extracellular Tat *in vitro* no longer proliferate to tetanus toxoid, [63], [64] Staphylococcal enterotoxin B, [65] or anti-CD3 monoclonal antibodies. [35], [66] In addition, neutralizing anti-Tat antibodies in the serum of HIV-infected individuals have been correlated with low viral loads and slower disease progression. [64], [67], [68], [69] [70] In view of this, Tat is currently being developed as both a prophylactic and therapeutic HIV vaccine in both animal models and in humans. [71], [72], [73], [74], [75], [76], [77] Ensoli and colleagues recently showed therapeutic immunization of HIV+ patients on antiretroviral therapy with Tat protein resulted in a durable anti-Tat humoral response. What is most striking is that individuals who developed anti-Tat antibodies demonstrated a normalization of T-cell phenotypes as well as increased CD4 and CD8 T-cell viability, proliferation and response to antigens. [75] Although CD127 was not analyzed in their study, the immune restoration evident in vaccinated individuals with anti-Tat antibodies could be explained at least in part by the recovery of IL-7 signaling. Anti-Tat antibodies generated through vaccination could neutralize soluble Tat protein in the serum and lymphoid tissues of patients and thereby allow recovery of CD127 on the surface of CD4 and CD8 T-cells. Indeed, we have also previously shown anti-Tat antibodies block Tat’s ability to down regulate CD127 on CD8 T-cells isolated from healthy individuals. [35] IL-7′s established roles in increasing cell viability through up regulation of Bcl-2, [78], [79] regulating peripheral T-cell number, [14], [15], [80], [81], [82] establishing T-cell memory, [18], [19], [20] and enhancing CD8 T-cell effector function [25], [26], [27], [31], [83], [84] could all explain the increase in cell viability, increase in CD4 T-cell number, increased percentage of central memory CD4 and CD8 T-cells as well as the increased expression of activation markers on CD8 T-cells and improved CD4 and CD8 T-cell responses to recall antigens demonstrated in Tat-vaccinated individuals. [75].

We show here that CD8 T-cells isolated from HIV+ individuals can re-express CD127 when purified and cultured *ex vivo*. This suggests existing CD8 T-cells present in HIV+ patients may not be irreversibly impaired and that a soluble factor or factors maintains suppression of CD127 *in vivo*. While a number of factors may be responsible for down regulating CD127 in HIV+ individuals, we demonstrate here that soluble Tat protein alone can maintain suppression of CD127 on the surface of CD8 T-cells isolated from HIV+ patients. This provides further evidence that Tat likely plays a major role in the suppression of CD127 *in vivo*. This in conjunction with our previous data showing Tat induced down regulation of CD127 results in impaired CD8 T-cell proliferation and cytolytic potential [35] and the apparent immune restoration documented by Ensoli and colleagues in patients undergoing therapeutic Tat vaccination [75] lends further support to the idea that soluble Tat protein likely contributes to poor lymphocyte responses and overall immune dysregulation in HIV+ patients.
